# Blood Flow Restriction Training in Nonspecific Shoulder Pain: Study Protocol of a Crossover Randomised Controlled Trial

**DOI:** 10.3390/sports11100197

**Published:** 2023-10-09

**Authors:** Kyriakos Pavlou, Christos Savva, Vasileios Korakakis, George M. Pamboris, Christos Karagiannis, George Ploutarchou, Antonis Constantinou

**Affiliations:** 1 Department of Health Sciences, European University Cyprus, 1516 Nicosia, Cyprus; g.pamboris@euc.ac.cy (G.M.P.); c.karayiannis@euc.ac.cy (C.K.); g.ploutarchou@external.euc.ac.cy (G.P.); an.constantinou@euc.ac.cy (A.C.); 2 Department of Health Sciences, Frederick University, 3080 Limassol, Cyprus; c.savva@frederick.ac.cy; 3 Department of Health Sciences, University of Nicosia, 1700 Nicosia, Cyprus; vkorakakis@hotmail.com

**Keywords:** BFR, blood flow restriction, shoulder pain, hypoalgesia, occlusion, pain reduction

## Abstract

“Nonspecific shoulder pain” encompasses various non-traumatic musculoskeletal shoulder disorders, diverging from diagnostic terminologies that refer to precise tissue-oriented clinical diagnosis. Blood flow restriction (BFR) training, involving partial arterial inflow and complete venous outflow restriction, has exhibited acute hypoalgesic effects primarily in healthy populations by increasing their pain thresholds. This study aims to examine whether a single BFR session with low-load exercises can alleviate pain perception among nonspecific shoulder pain patients. Conducted as a single-blind crossover randomised clinical trial, 48 adults (age range: 18 to 40) presenting with nonspecific shoulder pain will partake in two trial sessions. Random assignment will place participants into BFR or sham BFR groups and ask them to perform one exercise with BFR. Subsequently, participants will complete a shoulder girdle loading regimen comprising six exercises. The second session will involve participants switching treatment groups. Pain pressure thresholds (PPTs), shoulder pain and disability via the shoulder pain and disability index (SPADI), maximal voluntary isometric contraction (MVIC) of shoulder external rotators, pain during active abduction, and peak pain during shoulder external rotation will be evaluated using the numeric pain rating scale (NPRS). Immediate post-exercise assessments will include patient-perceived pain changes using the global rating of change scale (GROC) and participant-rated perceived exertion (RPE), employing a modified Borg’s scale (Borg CR10) post-BFR or sham BFR exercise session. Each session will encompass three assessment periods, and a combination of mixed-effect models and descriptive statistics will underpin the analysis. This protocol was approved by Cyprus National Bioethics Committee (ΕΕΒΚ/2023/48), and was registered in ClinicalTrials.gov (Registration number: NCT05956288). Conclusion: The anticipated outcomes of this study illuminated the acute effects of BFR training on pain perception within the context of nonspecific shoulder pain, potentially advancing strategies for managing pain intensity using BFR techniques.

## 1. Introduction

Shoulder pathologies are the third most common musculoskeletal condition encountered by health professionals in their day-to-day clinical practice, followed by low back and neck pain [1]. Notably, approximately 40–54% of patients report ongoing pain lasting 1–3 years [1], causing a significant economic burden on national healthcare systems and patients [2,3]. The pathologies associated with the development of shoulder pain are numerous; however, contemporary musculoskeletal shoulder pain research has emphasised the need to break free from diagnostic terminologies that refer to precise tissue-oriented clinical diagnosis [4]. The low validity and diagnostic accuracy of the special tests available to the clinician for shoulder joint evaluation [5,6,7,8] indicate the specific diagnosis as an elusive goal in clinical practice. In addition, the need to detach from diagnostic labels becomes even more intense if one takes into account the inability to correlate symptoms and clinical status with pathological findings in diagnostic tests [9,10,11]. Hence, for clinical practice, nonspecific shoulder pain is the preferred term as an umbrella term that includes non-traumatic musculoskeletal disorders of the shoulder.

Therapeutic exercise loading is the mainstay of conservative treatment and appears to be a powerful tool in the hands of clinicians to improve pain, mobility, and shoulder function in individuals with nonspecific shoulder pain [12]. Several mechanisms are thought to explain the benefits of exercise in nonspecific shoulder pain [13]. The most frequently mentioned neuromuscular mechanism suggests that strengthening the rotator cuff muscles inhibits pain [14]. Other mechanisms proposed are tissue factors (e.g., tendon remodelling, blood flow improvement, etc.), neuro-endocrine immunity (e.g., exercise-induced hypoalgesia, central and peripheral nervous system adaptations, etc.), and physiological mechanisms (e.g., improvements in self-efficacy and coping with pain, etc.) [13,15].

Blood flow restriction training (BFR) is a type of exercise with parallel partial restriction of the arterial inflow and complete venous outflow restriction in the muscle tissue that is usually combined with parallel execution of low-load resistance exercise loading. Blood flow restriction is achieved by using inflatable cuffs applied to both the upper and lower extremities [16,17]. During the last decade, the popularity of this technique seems to have grown rapidly due to its beneficial effects on increasing muscle strength and muscle hypertrophy in healthy adults [18,19,20] and clinical populations [21,22], as well as its acute hypoalgesic effect on the healthy population by significantly increasing the patient pain threshold [23,24,25]. However, to date, only one published study has examined the acute hypoalgesic effect of BFR training in a clinical population, showing beneficial adaptations in reducing pain in people with patellofemoral pain [26].

The primary objective of the present study is to evaluate whether a combination of a single acute BFR with a low-load exercise bout would reduce pain perception in patients with nonspecific shoulder pain and whether the potential hypoalgesia would be maintained after a 45 min physical therapy shoulder exercise loading session. The BFR exercise protocol will be compared to a sham BFR exercise protocol. We hypothesise that the participants in the BFR group would experience reduced pain perception and would be able to complete a scapula and rotator cuff muscle loading program with reduced pain.

## 2. Materials and Methods

### 2.1. Study Design

**Figure 1:**
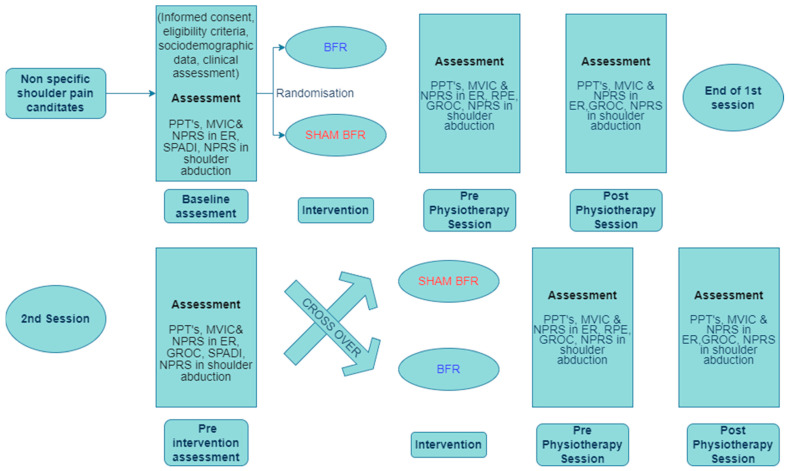
Schematic diagram of the study design. Abbreviations: BFR, blood flow restriction; ER, external rotation; GROC, global rating of change scale; MVIC, maximal voluntary isometric contraction; NPRS, numeric pain rating scale; PPTs, pain pressure thresholds; RPE, rating of perceived exertion; SPADI, shoulder pain and disability index.

This crossover design randomised controlled clinical trial will include two intervention arms (BFR and sham BFR) with three assessment time points (baseline, immediately after BFR training, and at the end of the shoulder girdle exercise loading session) (Figure 1). Outcome measures assessment during the first (DAY 1) and the second session (DAY 2) will be performed by a blinded independent assessor to the intervention (sham BFR or BFR) to avoid the risk of “data collection ascertainment bias”.

All participants will be blinded to the group allocation to avoid the risk of “expectation bias” and “patient ascertainment bias” [27]. To achieve this, they will be informed that the effect of two different BFR occlusion protocols on pain perception after a low-load resistance exercise bout will be evaluated. The physiotherapists performing the interventions (BFR or Sham BFR) will not be blinded; however, they will not participate in the outcome assessment and will be blinded to the recorded values.

All participants will have an introductory baseline assessment. They will be asked to fill out questionnaires on sociodemographic characteristics, symptoms, comorbidities, and functional limitations. Subsequently, measurement of pain pressure thresholds (PPTs), administration of the Shoulder Pain and Disability Index (SPADI), measurement of maximal voluntary isometric contraction (MVIC), assessment of pain during active abduction, and maximal pain in shoulder external rotation on the numeric pain rating scale (NPRS) will be recorded. Then, participants will be randomly allocated to one of the two intervention groups (BFR and Sham BFR) and will be asked to perform one exercise with BFR. Immediately after the BFR session, they will be asked to evaluate the level of difficulty or exertion experienced during the BFR or Sham BFR exercise using the Borg CR1O scale [28]. Simultaneously, measurement of PPTs, MVIC, and maximal pain during external rotation, pain during active shoulder abduction, and perception of improvement or worsening of symptoms will be recorded. Finally, the participants will be asked to perform the shoulder girdle loading protocol, and a final assessment of the same outcome measures will be performed.

The participants will be crossover randomised, and the testing sessions will be at least 72 h apart to ensure that their clinical status (pain and other symptoms) has not changed. More specifically, they will be asked: “Has your clinical situation changed since last time?” If their answer is no, they will continue with the rest of the procedure; otherwise, they will be excluded from the second session, and this will be reported. This RCT is registered in ClinicalTrials.gov (Registration number: NCT05956288), and the study was designed according to the Consolidated Standards of Reporting Trials (CONSORT) guidelines for randomised clinical trials [29].

### 2.2. Sampling Method

Participants will be recruited through advertisements at local universities, physical therapy clinics, hospitals, and social media.

### 2.3. Participants and Sample Size

Adults presenting with nonspecific shoulder pain will be recruited using the following inclusion criteria: (1) age from 18 to 40 years, considering that the prevalence of degenerative rotator cuff tendinopathy increases from the age of 40 and above [30], (2) shoulder pain intensity greater than 4/10 on the NPRS [24] when performing active glenohumeral abduction and/or isometric resistance to glenohumeral external rotation at 0° of abduction, and (3) duration of symptoms greater than 3 weeks (to avoid an inflammatory component in the symptoms).

Participants will be excluded if they present any of the following criteria: (1) positive drop arm test (important indication for rotator cuff tear) [31], (2) cervical radiculopathy by presenting a positive specific Spurling test or neurological symptoms in the upper limb [32], (3) passive deficit in the active range of motion (AROM) at more than two levels of motion, where according to the literature it is an indication of a frozen shoulder [33], (4) body mass index (BMI) ≥ 30 [34], (5) previous shoulder surgery, (6) previous humerus fracture, (7) cancer, (8) rheumatic diseases, (9) systemic pathologies (e.g., diabetes, rheumatic disorder, fibromyalgia), (10) history of previous neurological disease, (11) history of thrombosis, (12) cardiovascular pathology, (13) neurological disease, (14) long-term use of corticosteroids, (15) injury to or surgery in the shoulder girdle less than 1 month from recruitment, (16) hypertension (systolic blood pressure greater than 140 mmHg and diastolic blood pressure greater than 90 mmHg), (17) anti-inflammatory drug treatment in the last 2 weeks, (18) injectable therapy (last 3 months), (19) previous experience/knowledge of BFR training [35], and (20) inability to write and read Greek.

The primary outcome used to calculate the required sample size using G* Power (v 3.1) software was pain. The sample size calculation was based on a previous study that reported an effect size of d = 0.6 for differences in the NPRS between groups after the BFR exercise [26]. To achieve 95% power at an alpha level of 0.05, 20 patients per intervention for 3 assessment periods of outcome measures are required. Given the crossover design of the study and the possibility that the status of the participants will change between the first and the second intervention, it is estimated that there will be a dropout of 20%, and thus, it is proposed to recruit 24 participants per group into the study, or 48 participants in total.

### 2.4. Randomisation and Blinding

Participants meeting the predetermined inclusion criteria will be randomly allocated to the BFR group (n = 24) or the sham BFR group (n = 24). For the randomisation of the participants, the “block randomisation” method will be applied through the randomisation website (http://www.randomization.com) by an external researcher, who will not have any contact with the participants throughout the study [27,36]. To ensure a balance in sample size between groups over time, we will randomise participants using a block randomisation method (blocks of 4). Random codes will be sealed in opaque envelopes and delivered to the physical therapist, who will undertake the intervention by an administrative assistant who will not be involved in the study [26].

### 2.5. Interventions

Prior to the main part of the intervention, all patients will be familiarised with the exercise protocol. The only difference between the two intervention groups will be the percentage of BFR cuff pressure. The total duration of the two interventions, including the measurements, will be approximately 1 h.

#### 2.5.1. Sham Group

In the sham BFR group, the cuff will be placed firmly around the proximal deltoid and automatically inflated until there is enough room for two fingers between the skin and the cuff. This will be automatically applied via the MAD-UP^®^ system, which has been designed based on published research and does not promote tissue adaptation (i.e., neurological, structural) [37].

#### 2.5.2. BFR Protocol

The vascular occlusion pressure needed for the complete occlusion of the upper extremity blood flow will be measured at rest, with the participant relaxed in the standing position [38], by placing a 6 cm wide and 60 cm long cuff on the more proximal part of the affected upper limb using an automatic personalised tourniquet system designed to automatically calculate limb occlusion pressure (MAD-UP Pro system, Angers, France) with clinically acceptable accuracy and high reliability [39,40]. Both intervention groups will perform the same exercise protocol. The limb occlusion pressure will be set at 50–60% of the complete occlusion pressure for the intervention group [35].

#### 2.5.3. Exercise Protocol

All participants will perform biceps curls with a dumbbell from a standing position using BFR or sham BFR. The rhythm of the exercise will be paced using a metronome (60 bpm), and the biceps curls for each muscle contraction mode (concentric or eccentric) will last 2 s [41]. The initial resistance load will correspond to 5% of each participant’s body weight (±0.250 kg). Participants will be asked to perform 4 sets of bicep curls. The first set will consist of repetitions until failure (inability to follow the pace of the metronome, inability to complete the contraction on full range, or inability to perform an additional contraction), followed by 3 sets of 15 repetitions with a 30 s rest between the sets while the cuff remains inflated [25]. Therapists will encourage the participants to reach their maximum possible number of repetitions and inform them of the importance of completing all four sets. The participants’ inability to complete all set repetitions or to follow the pace of the metronome will indicate a resistance load reduction of 0.5 kg [42]. Immediately after the execution of the biceps curl exercise, the cuff will be deflated and removed for both groups.

#### 2.5.4. Muscle Loading Protocol of the Shoulder Girdle after the BFR Application

Five minutes after the execution of the biceps curl exercise, each participant will receive a structured physiotherapy session consisting of six therapeutic exercises targeting the rotator cuff and scapula muscles. Initially, the participants will be asked to perform two warm-up exercises that activate several muscle groups of the shoulder girdle: (1) a bear hug exercise from a standing position using a pulley, and (2) a low row exercise using a pulley [43] (Supplementary File S1). Both exercises will be performed in 4 sets of 12 repetitions at a ratio of 2 s concentric to 2 s eccentric contraction paced using a metronome. Calculation of the load will be performed using the 2 in-reserve method [22]. Then, the participants will proceed to the main part of the training, which will include the following exercises: (1) shoulder press (closed grip) from a seated position, (2) internal rotation of the shoulder, (3) external rotation of the shoulder with a towel under the armpit (20 o–30 o relative shoulder abduction position), and (4) shoulder flexion. Apart from the shoulder press exercise, the other three exercises will be performed in the standing position [44,45] (Supplementary File S1). A modified pain monitoring approach will dictate the loading, so the patient will report a maximum of 4/10 during familiarisation or training [26,42,44,45]. The participants will start with the shoulder press exercise, performing four sets with a load corresponding to 10% of their body weight. All four sets will be performed until failure, with a one-minute rest between sets [26,42]. For the remaining exercises, the load will be set at 5% of their body weight. Likewise, all four sets will be performed until failure, with a one-minute rest between sets. The physiotherapy session will be performed without the use of BFR.

### 2.6. Data Collection

Data collection will take place on the premises of the European University Cyprus before the beginning of the interventions (baseline), immediately after the biceps curl exercise with BFR and sham BFR (pre-loading), and after the end of the shoulder girdle training session (post-loading). Then, the participants will undergo a 72-h wash-out period to avoid any potential carryover effects before the intervention crossing over.

The main outcome measures will be: (a) identification of PPTs (three local and three remote sites), (b) maximal pain during MVIC of shoulder external rotators, and (c) MVIC of shoulder external rotators.

#### 2.6.1. Pressure Pain Threshold Assessment

PPTs will be assessed using a handheld mechanical pressure algometer (Wagner Instruments, Riverside, CT, USA) with a 1 cm^2^ rubber disk attached to the pressure gauge by applying the probe perpendicularly to the skin at a pressure rate increase of approximately 1 kgf per second. One assessor blinded to the group allocation will obtain PPT measurements with the participants in long sitting with both arms resting on the plinth. The participants will be instructed to report the moment when the pressure sensation changes into slightly unpleasant pain. PPT assessment sites will be located, marked, and evaluated over (1) the upper trapezius (middle of the distance between the acromion and spinous process A7), (2) the supraspinatus (muscle belly muscle just below the middle of the spine of the scapula), (3) the middle deltoid (middle distance from the acromion and the epiphysis of the muscle), (4) the abductor pollicis longus (midway of muscle belly), (5) the quadriceps (midway between anterior superior iliac crest and superior pole of patella), and (6) the tibialis anterior muscle (muscle belly, midway between tibial tuberosity and ankle) [23,46,47,48]. The average pressure, in kilograms, of the three trials will be used for data analysis. The order of scoring points will be randomised between patients with 30 s intervals between the three trials. PPT assessment using a handheld algometer has been reported to be a valid and reliable procedure [49,50,51].

#### 2.6.2. Maximal Pain and Strength in Isometric Shoulder External Rotation

The MVIC of shoulder external rotation will be assessed using a handheld dynamometer (Kinvent, Montpellier, France) from a standing position, with the legs apart at shoulder height, the hips and knees in slight flexion, and the elbow on the examined upper limb at 90° flexion with the thumb facing up. A towel will be placed at the height of the elbow to slightly abduct the shoulder at 20–30° [52].

The participants will be asked to apply a progressive MVIC of the external rotators for 5 s, instructing them to “push, push…push…push…push…relax” in a monotone voice [53]. If the participant cannot exert more force due to pain, the MVIC will be calculated until that point. The examiner will hold the handheld dynamometer in a position to achieve perpendicular alignment with the styloid process of the ulna [52]. The handheld dynamometer has demonstrated perfect test-retest reliability with an interclass correlation coefficient (ICC) of 0.92–0.98 and very good to perfect inter-examiner reliability with an ICC of 0.84–0.96 for assessing the MVIC of shoulder external rotation [52,54]. The maximum value from three successful trials separated by a minimum 30-s break (trials with more than 15% difference will be discarded) will be used for the statistical analysis [52]. In addition, during the test, the participants will be asked to rate their pain based on the NPRS (0–10).

#### 2.6.3. Secondary Outcomes

The following secondary outcomes will also be assessed: (a) pain during active shoulder abduction (0 to 180°) in the standing position using the NPRS, (b) patient-perceived pain change using a global rating of change scale (GROC) based on a 7-point Likert scale ranging from “Much better—a very important improvement” to “Much worse—an important aggravation” [55] (not applicable at baseline), and (c) the participants’ rating of perceived exertion (RPE) using a modified Borg’s scale (Borg CR10) immediately following the BFR or sham BFR exercise session. The scale has been shown to be a valid alternate tool for estimating exertion and intensity levels during resistance training [56]. A printed version of the modified scale will be presented to the participants, and it will be explained to them that a very hard session would have a rating of ≥7 out of 10 and a rating of 10 means that they were giving maximal effort and could not exert themselves any further. Accordingly, a light session may have a rating of Borg ≤ 2, a moderate session may have a rating of Borg >2–<5, and a hard session may have a rating of Borg ≥5–<7. (d) The Shoulder Pain and Disability Index (SPADI) questionnaire assesses the pain and difficulty experienced by the participant in daily activities of the upper extremity [57]. This questionnaire consists of two parts. During the first part, the participants will be evaluated on a scale of 0–10 on the pain they experience in daily activities, and during the second part, the participants will be evaluated on the difficulty they experience in performing the various daily activities (0–10). This questionnaire has been translated and cross-culturally adapted for the Greek-speaking population [58].

### 2.7. Statistical Analysis

Descriptive statistics will be used to present and describe participant characteristics (values will be presented as mean and standard deviation or median and interquartile range).

The residuals of each variable/model will be tested for normality by visual inspection of frequency histograms and Q–Q plots [59] or by using the Shapiro–Wilk test [60]. Mixed-effects models will be constructed considering the primary outcome measures (PPTs, MVIC, and maximal pain during external rotation) using participant random effects for all assessment time points. Fixed effects will include the day of assessment (DAY 1 or DAY 2), the two treatment groups (BFR and sham BFR), the time points of assessment of the outcome measures, and all interactions. Additionally, we will model parameter estimates for variables associated with pain responses, such as age, height, and condition chronicity. If a statistically significant effect or interaction is found, we will perform a post hoc test using Tukey’s adjustment for multiple comparisons. Statistical analyses will be conducted using JMP (v.16.0, SAS Corporation, Cary, NC, USA), and the statistical significance will be set at α = 0.05.

### 2.8. Withdrawal of Individual Participants

Participation in this study is completely voluntary, and participants do not have to give a reason if they do not wish to participate. If they decide to take part, they are free to change their mind and withdraw from research at any time without a reason. Unexpected, related, serious adverse events will be reported to the Research Ethics Committee within 15 days of a research team member becoming aware of the event.

## 3. Discussion

Nonspecific shoulder pain, as mentioned above, brings about a substantial economic impact not only on the national health systems but also on the patients themselves, leading them to pain chronicity, increasing in parallel the risk of adverse consequences such as decreased participation and quality of life, absenteeism at work, and early retirement [1,2,3,61]. Consistent with the available literature, a rehabilitative program that includes strengthening exercises for the scapula and rotator cuff muscles appears beneficial for improving individuals’ function and quality of life with nonspecific shoulder pain [12,62,63,64]. Despite this, up to 30% of individuals still present with pain and disability after rehabilitation interventions due to the continuation of the pain, which leads to dissatisfaction and difficulty in completing such exercise programs [61]. Thus, we aim to investigate whether a single BFR training session with a low load may be used to reduce pain and provide a window of opportunity for clinicians to optimally load otherwise painful tissues and joints.

The specific type of study was chosen since it was considered the most appropriate to examine any direct acute effect with regard to pain perception following BFR training, since the result of the intervention is compared within the same participant, and each participant serves as his own control without ethical issues arising from a placebo group. Another advantage of this design is the reduction of inter-participant variability by comparing the two interventions. Finally, the crossover design provides higher statistical power, even with a smaller number of participants, than simple randomised controlled clinical trials [65].

## 4. Limitation

The main limitation of the present study is that performing multiple tests at three different times in each session may aggravate pain in people with nonspecific shoulder pain. Thus, taking into consideration that aggravation of the symptoms may be affected by the order in which each test will be executed, we decided to randomise the order in which each test will be performed. An additional limitation of the present study is the non-inclusion of post-surgery patients in order to minimise the variability in the study population.

## 5. Conclusions

The present study will be the first to examine the possible acute effects of BFR training on pain perception in participants with nonspecific shoulder pain. It may add new insights into controlling pain intensity using the BFR technique or a pain pre-conditioning approach in rehabilitation. In addition, if BFR proves effective, it may be the starting point for designing further studies that examine the long-term hypoalgesic effect in clinical populations with musculoskeletal conditions.

## Data Availability

Pseudonymised quantitative participant data will be made available in a public repository after data publication.

## Supplementary Materials

The following supporting information can be downloaded from https://www.mdpi.com/article/10.3390/sports11100197/s1, Supplementary File S1: Muscle loading protocol of the shoulder girdle.
