# Supplementary material for: Blood Flow Restriction Training in Nonspecific Shoulder Pain: Study Protocol of a Crossover Randomised Controlled Trial
Source: Sports (Basel). 2023 Oct 9;11(10):197. doi: 10.3390/sports11100197 (PMC10610701; doi:10.3390/sports11100197)
Supplement: Supplementary file 1 [file sports-11-00197-s001.zip › sports-2618243-supplementary.pdf]

Supplementary File S1: Muscle loading protocol of the shoulder girdle

1. Low row	
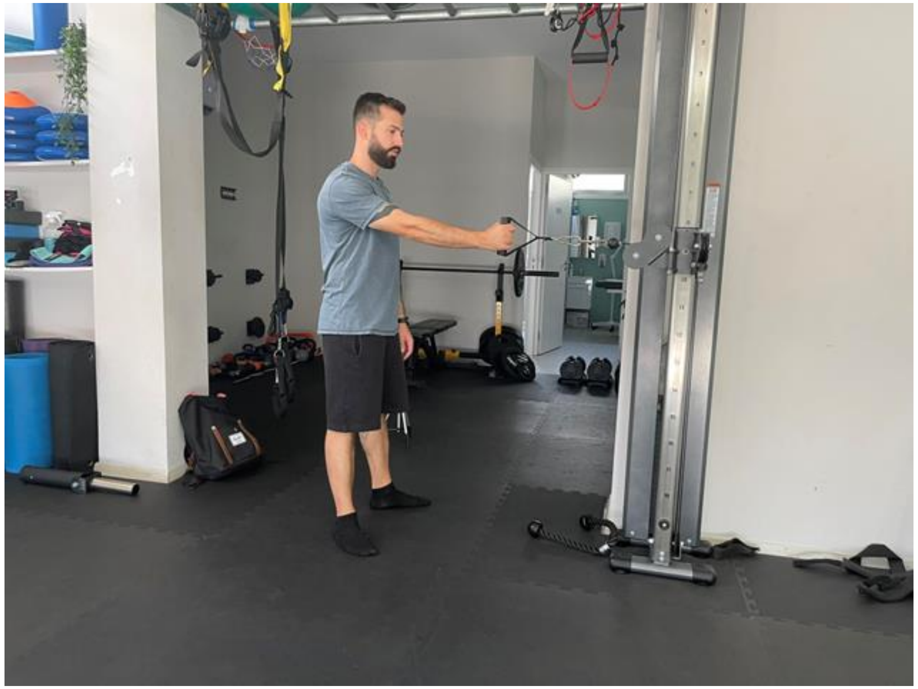<br>Start position	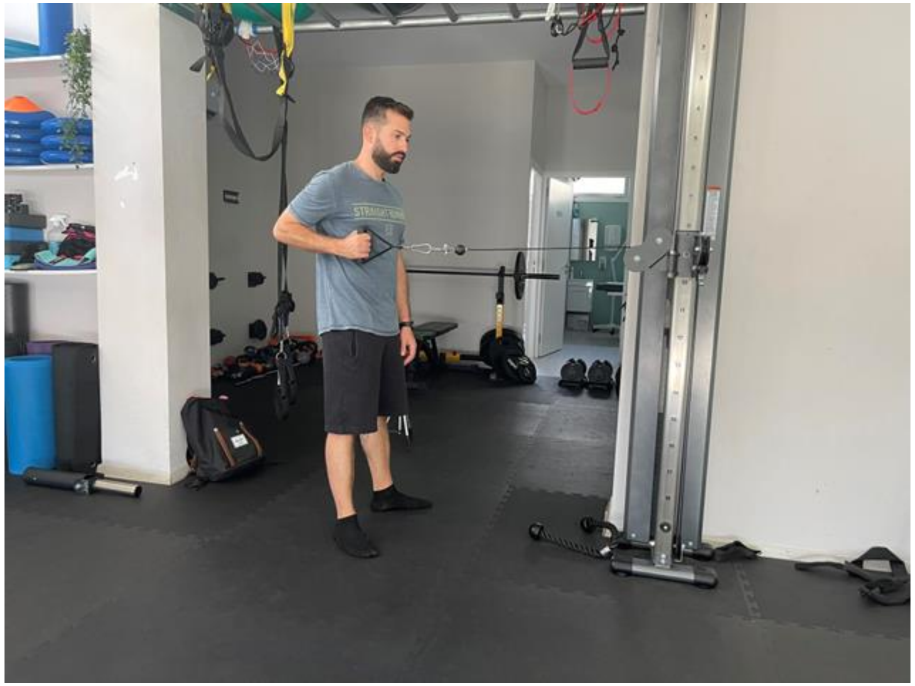<br>End position

2. Bear hug

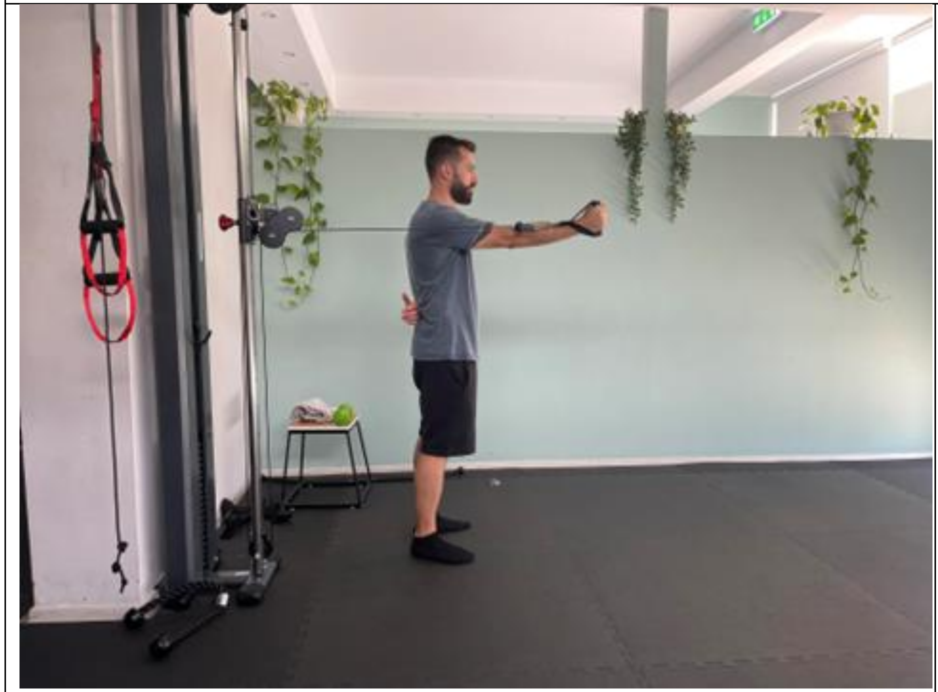

Start position

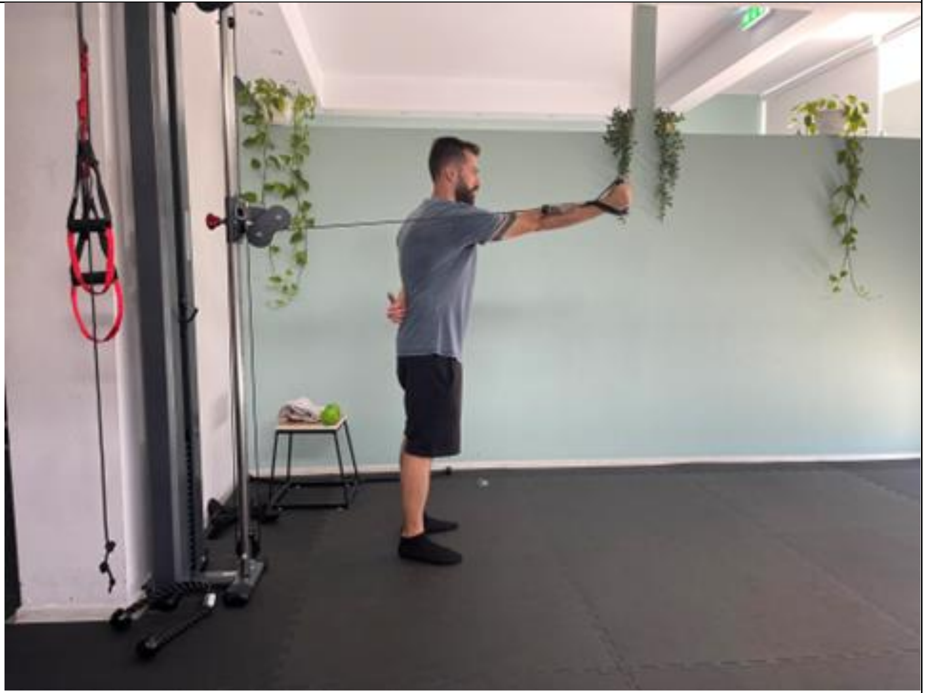

End position

3. Shoulder press

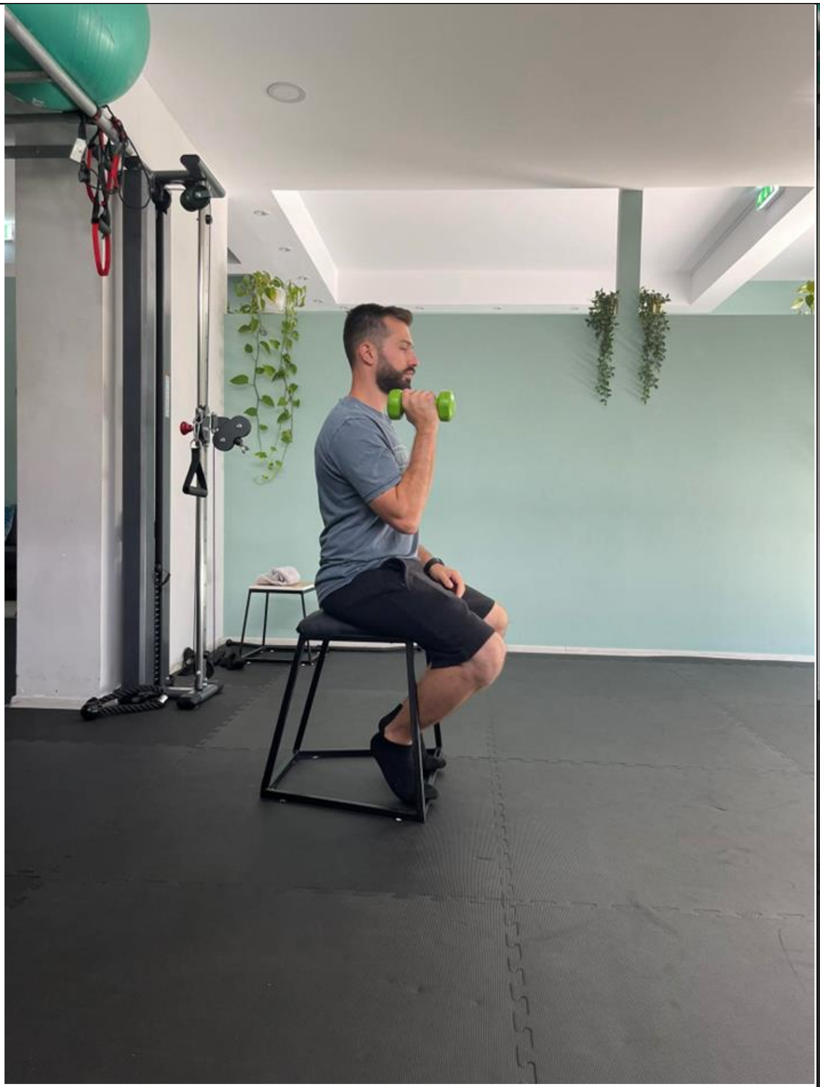

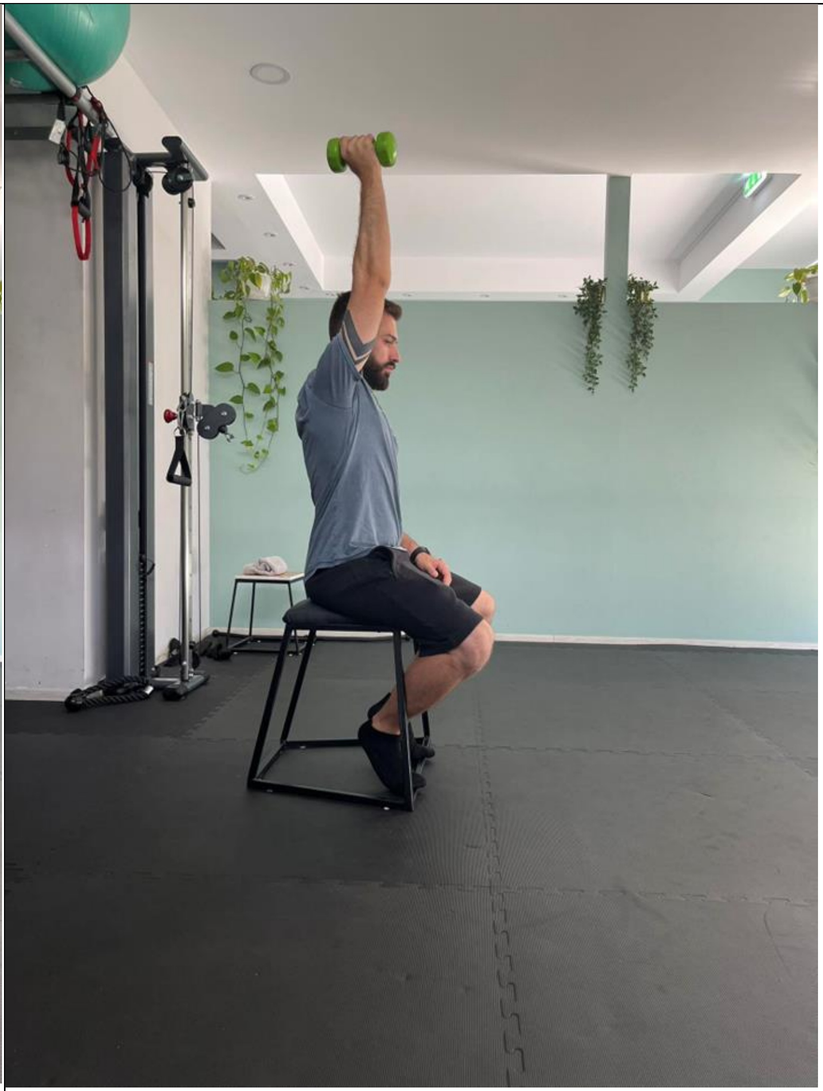

Start position	End position
4. Shoulder flexion	

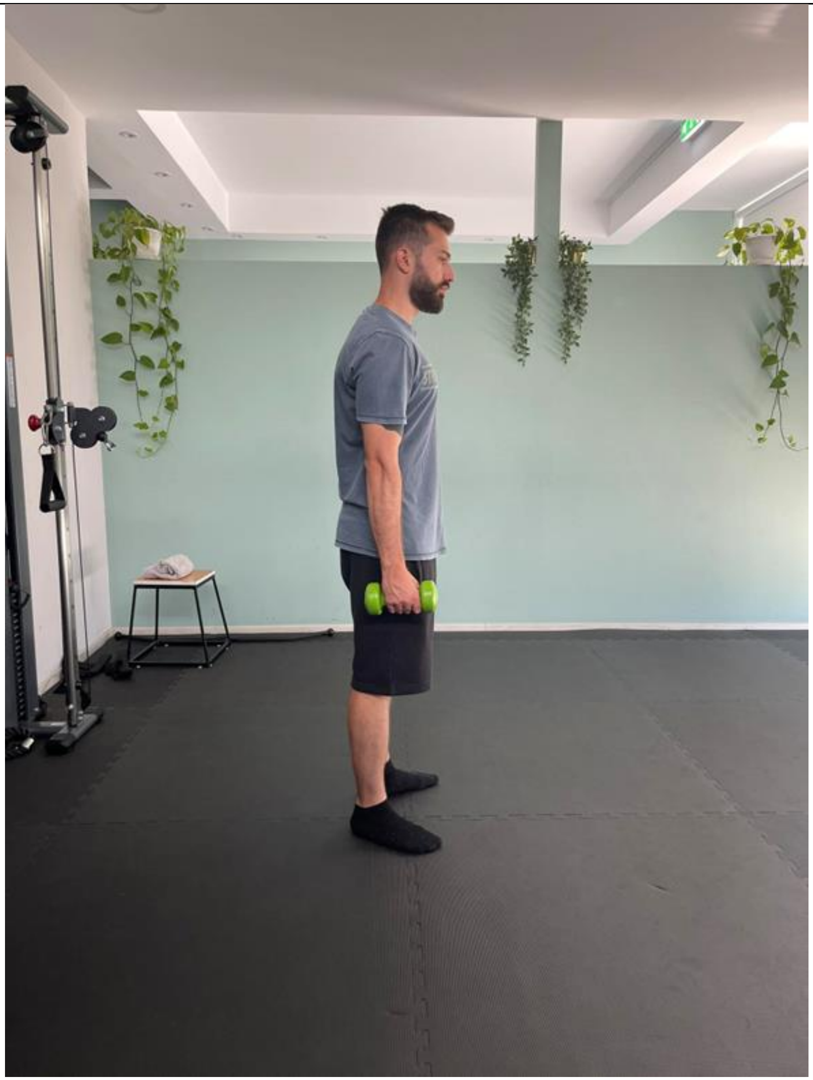<br>Start position	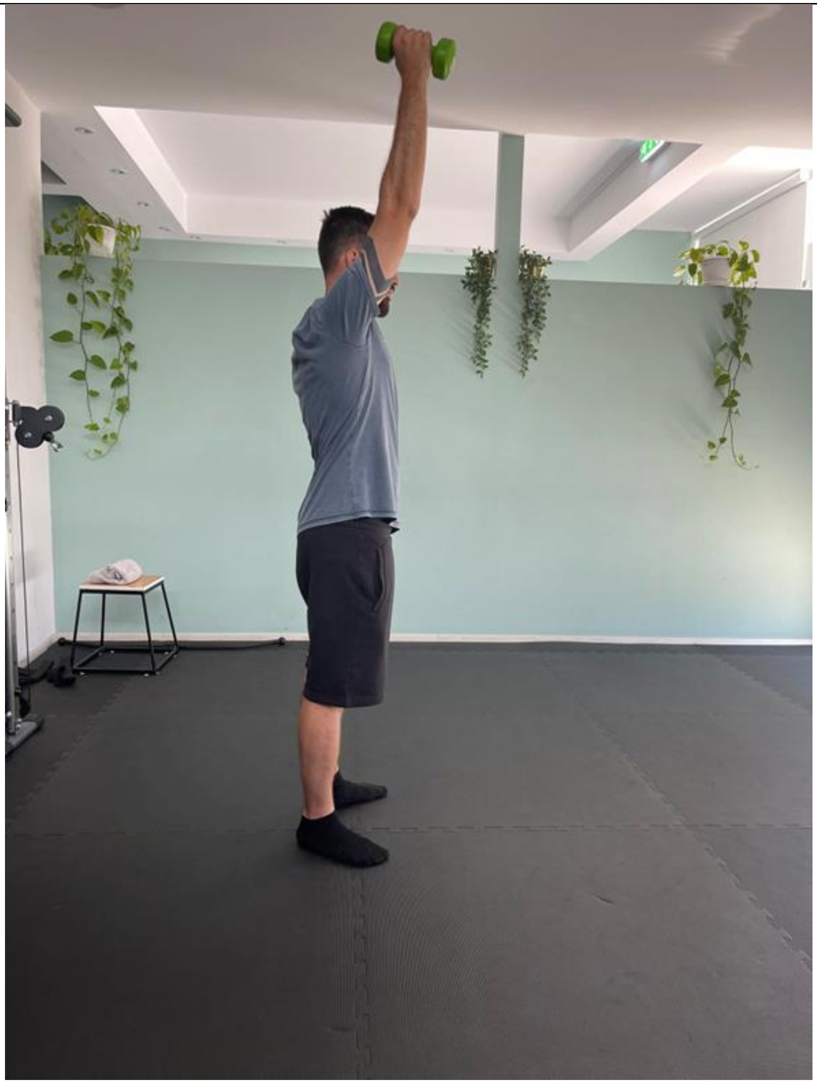<br>End position
5. Shoulder external rotation	

<table>
<tr>
<td>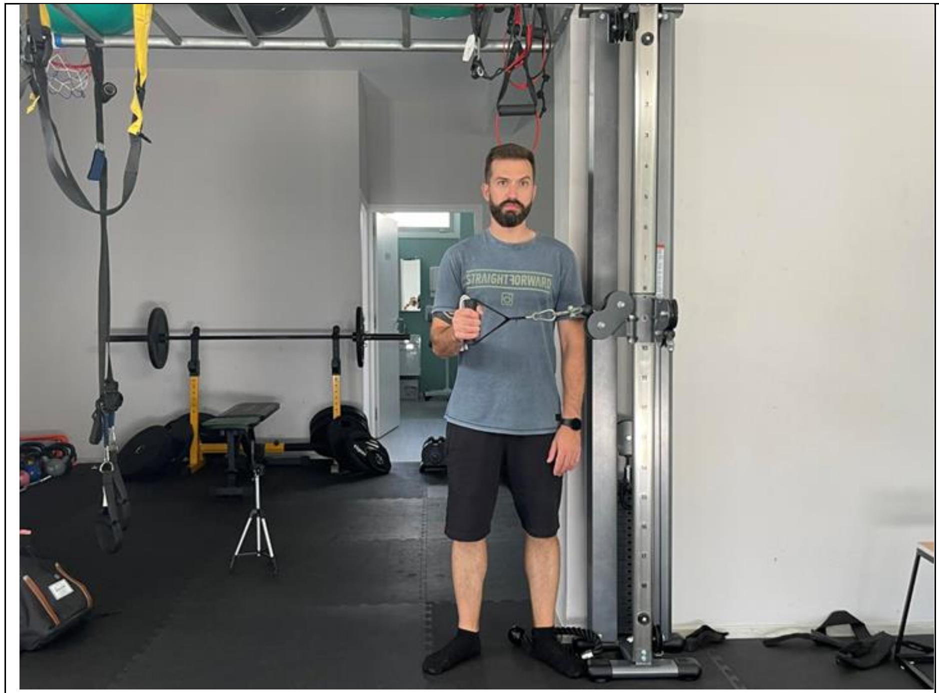

Start position</td>
<td>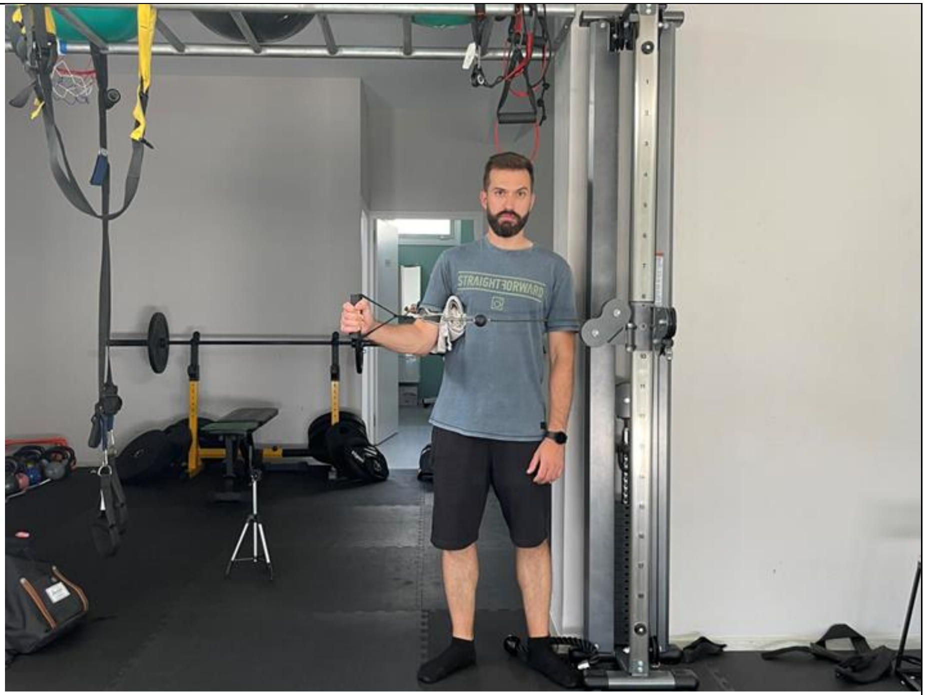

End position</td>
</tr>
<tr>
<td colspan="2">6. Shoulder internal rotation</td>
</tr>
</table>

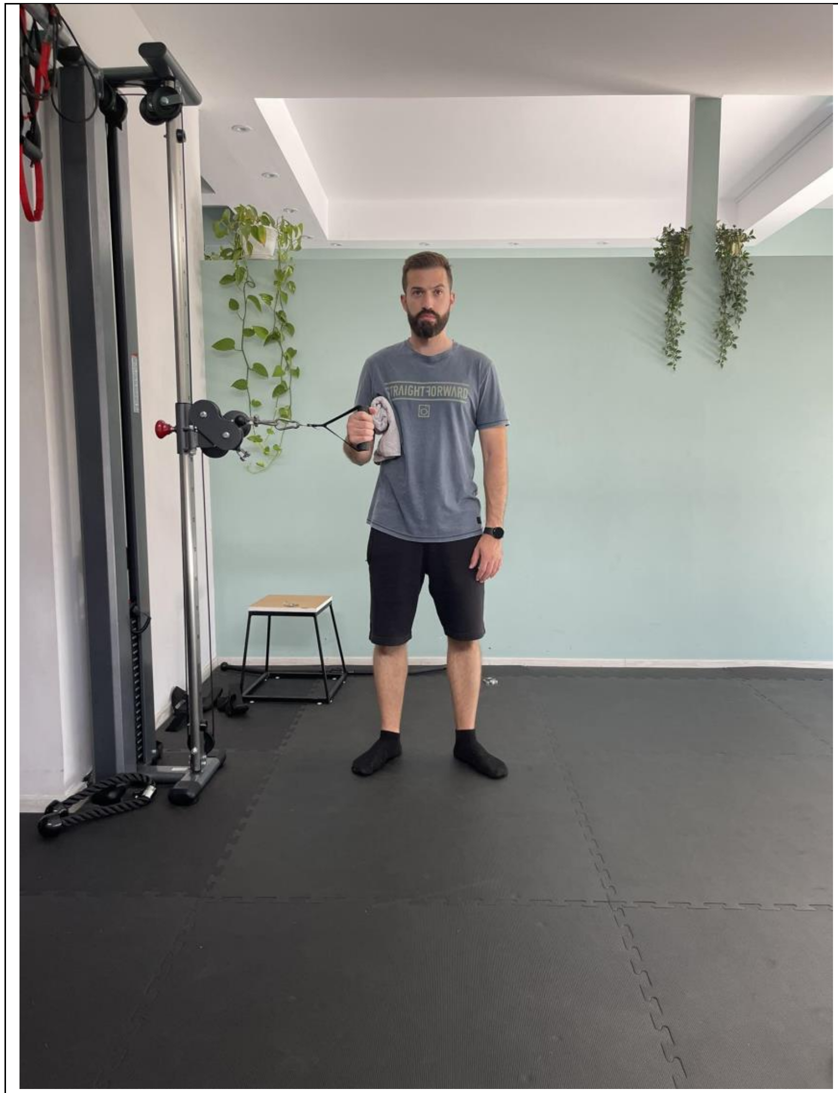	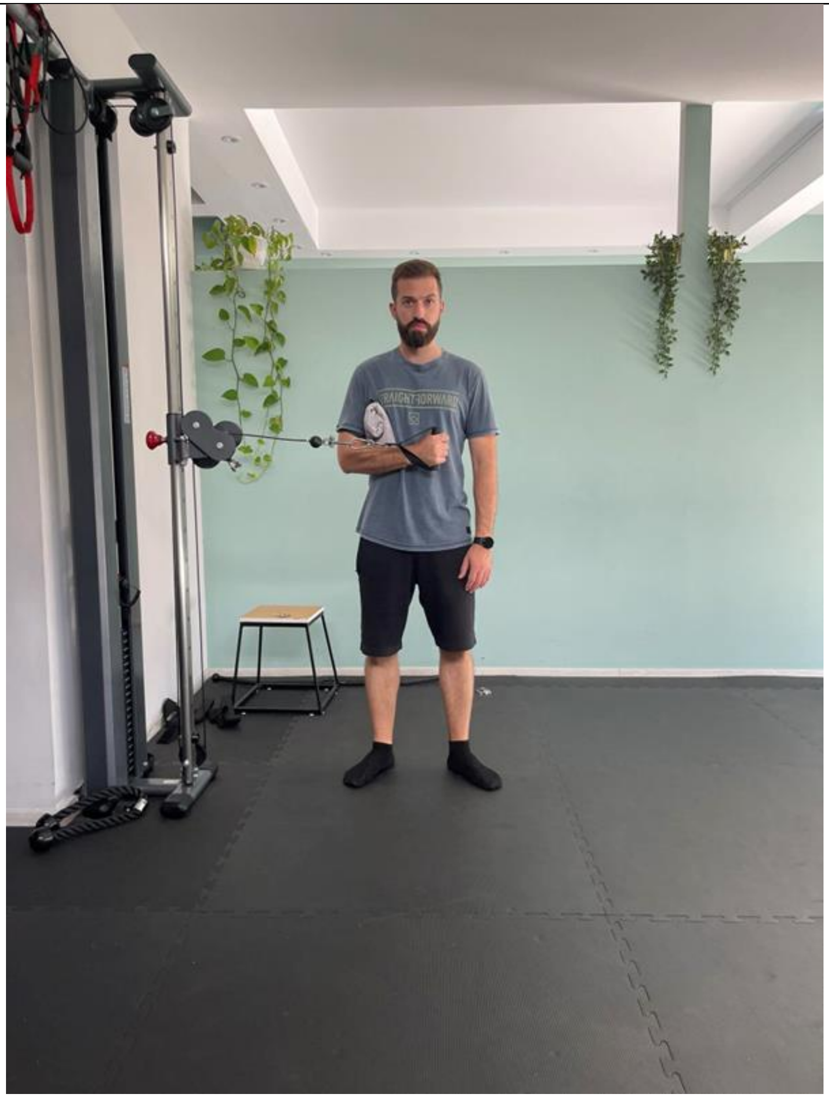
Start position	End position
